# Migratory Wave due to Conflicts: Risk of Increased Infection From Zoonotic Diseases

**DOI:** 10.1155/tbed/5571316

**Published:** 2025-01-30

**Authors:** Sina Salajegheh Tazerji, Phelipe Magalhães Duarte, Rasha Gharieb, Lukasz Szarpak, Michal Pruc, Md. Tanvir Rahman, Alfonso J. Rodriguez-Morales, Muhammad Furqan Ilyas, Maria de Nazaré Santos Ferreira, Yashpal Singh Malik, Roozbeh Kalantari, Ava Shahrokhabadi, Niloofar Jafari, Fatemeh Shahabinejad, Yasaman Maleki, Sina Montajeb, Roya Mehrpouya, Hadis Ahmadi, Bita Vazir, Farrokhreza Kabir, Abdul Rehman, Zahra Elmi, Pouneh Hajipour, Hesham R. El-Seedi, Wolfgang Eisenreich, Awad A. Shehata

**Affiliations:** ^1^Department of Clinical Sciences, Faculty of Veterinary Medicine, Islamic Azad University, Tehran, Iran; ^2^Young Researchers and Elites Club, Science and Research Branch, Islamic Azad University, Tehran, Iran; ^3^Postgraduate Program in Animal Bioscience, Federal Rural University of Pernambuco (UFRPE), Recife 52171-900, Pernambuco, Brazil; ^4^Department of Zoonoses, Faculty of Veterinary Medicine, Zagazig University, Zagazig 44511, Egypt; ^5^Department of Clinical Research and Development, LUXMED Group, Warsaw, Poland; ^6^Institute of Medical Science, Collegium Medicum, The John Paul II Catholic University of Lublin, Lubin, Poland; ^7^Henry JN Taub Department of Emergency Medicine, Baylor College of Medicine, Houston, Texas, USA; ^8^Department of Public Health, International European University, Kyiv, Ukraine; ^9^Department of Microbiology and Hygiene, Faculty of Veterinary Science, Bangladesh Agricultural University, Mymensingh 2202, Bangladesh; ^10^Clinical Epidemiology and Biostatistics, School of Medicine, Universidad Científica Del Sur, Lima, Peru; ^11^Gilbert and Rose-Marie Chagoury School of Medicine, Lebanese American University, Beirut, Lebanon; ^12^Department of Animal Science, University of Sargodha, Sargodha, Pakistan; ^13^Guru Angad Dev Veterinary and Animal Sciences University, Ludhiana 141004, Punjab, India; ^14^Department of Clinical Sciences, Faculty of Veterinary Medicine, Islamic Azad University, Shahrekord, Iran; ^15^Department of Clinical Sciences, Faculty of Dentistry Medicine, Hormozgan Medical School, Bandar Abbas, Hormozgan, Iran; ^16^Department of Clinical Sciences, Faculty of Medicine, Kerman Medical School, Kerman, Iran; ^17^Endocrinology and Metabolism Research Center, Kermanshah University of Medical Sciences, Kermanshah, Iran; ^18^Diagnostic Laboratory Sciences and Technology Research Center, Shiraz University of Medical Sciences, Shiraz, Iran; ^19^Department of Clinical Sciences, Faculty of Veterinary Medicine, Tehran University, Tehran, Iran; ^20^Department of Clinical Sciences, Faculty of Veterinary Medicine, Kazeroun Branch, Islamic Azad University, Kazeroun, Iran; ^21^Department of Clinical Sciences, Faculty of Veterinary Medicine, Karaj Branch, Islamic Azad University, Karaj, Iran; ^22^Department of Basic Sciences, Faculty of Veterinary Medicine, Science and Research Branch, Islamic Azad University, Tehran, Iran; ^23^Department of Epidemiology and Public Health, University of Veterinary and Animal Sciences Lahore, Lahore 54000, Pakistan; ^24^Young Researchers and Elite Club, Faculty of Veterinary Medicine, Babol Branch, Islamic Azad University, Babol, Iran; ^25^International Research Center for Food Nutrition and Safety, Jiangsu University, Zhenjiang 212013, China; ^26^Department of Chemistry, Faculty of Science, Islamic University of Medinah, Medinah 42351, Saudi Arabia; ^27^Department of Chemistry, TUM School of Natural Sciences, Bavarian NMR Center (BNMRZ), Structural Membrane Biochemistry, Technical University of Munich, Garching 85748, Germany

**Keywords:** ecosystem, One Health, pathogens, risk, wars, zoonoses

## Abstract

Wars have devastating effects on all the components of the One Health approach: humans, animals, and ecosystems. Wars and the resulting migratory waves massively disrupt normal animal health services and surveillance. Among other consequences, they adversely impact the early detection, prevention, and control of animal diseases. Uncontrolled movement of animals or their undisposed carcasses, the destruction of wildlife habitats, and the increased interface between humans, wildlife, and domestic animals contribute to uncontrolled transmission and spread of zoonotic pathogens from animals to humans. In the last millennium, zoonotic diseases such as the “Black Death” were triggered by devastating wars and led to the deaths of a large fraction of the human population. However, also recent and ongoing wars carry the risk of an uncontrollable increase in zoonotic diseases. The most significant zoonotic diseases reported during the recent wars are African swine fever, highly pathogenic avian influenza, rabies, leptospirosis, and brucellosis, as well as foodborne and waterborne zoonotic diseases. Indeed, alarming rates of infections by antimicrobial-resistant pathogens such as *Mycobacterium tuberculosis* go along with wars, as seen in the current Ukraine–Russia conflict. Considering human migration, foodborne and waterborne zoonotic diseases are key health threats for refugees due to the consumption of unsafe food, lack of safe water, and disruption of the water supply and sanitation system. This review summarizes the potential factors and some data associated with the increased risk of zoonotic disease emergence and transmission during recent and ongoing conflicts.

## 1. Introduction

Zoonotic diseases, characterized by their ability to be transmitted from animals to humans, have persistently presented substantial risks to worldwide public health. Public health professionals and academics must pay close attention to these diseases, as they can cross species barriers. The challenges in predicting the emergence and dissemination of zoonotic illnesses are compounded by their erratic behavior and intricate patterns, as elucidated in the research conducted by Sikkema and Koopmans [1]. The inherent unpredictability of certain phenomena can occasionally result in rapid global outbreaks. Failure to promptly execute preventive measures or interventions may lead to the emergence of severe pandemics, which can have significant societal and economic consequences on a worldwide scale [1]. Alterations in human social processes, whether gradually or abruptly, such as transformations in the meat business or military conquests, can engender zoonotic pandemics and novel infectious diseases in humans.

**Figure 1:**
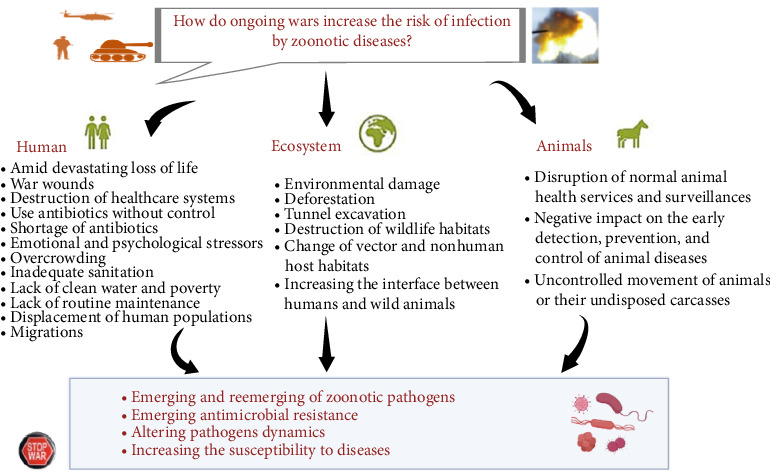
Potential impacts of wars on the emergence of diseases. The figure was generated by BioRender.

Amidst the political and social disruptions in countries affected by severe conflicts and wars, a significant and concealed problem arises: the increasing threat of zoonotic diseases, which are exacerbated by the patterns of migration resulting from the ongoing conflicts. The movement of substantial populations in response to upheaval results in an inadvertent outcome: ecosystems experience disruption and the demarcation between urban regions and wildlife habitats becomes increasingly indistinct. The act of intermingling not only threatens the ecological equilibrium, but also creates opportunities for the emergence and dissemination of new illnesses. The combination of human migration, ecological disturbance, and habitat overlap creates a suitable environment for transmitting illnesses from animals to humans, posing a significant health risk that may persist after resolving the conflict. In the present review, we will shed light on how the ongoing wars increase the risk of infection by zoonotic diseases (Figure 1).

## 2. Exposure to New Pathogens From Different Regions

In the aftermath of the recent wars, a significant number of citizens have been compelled to seek asylum in other regions. The escalating mobility of human populations has led to a heightened apprehension over the dissemination of infections across different geographical zones. When significant populations experience displacement, there exists an increased susceptibility not only to the direct transmission of diseases carried by humans, but also to a possible upsurge in zoonotic diseases [2]. Migration is a fundamental element of the human experience, marked by the displacement of persons from one geographical area to another. When analyzing regions embroiled in conflict, the complexities surrounding migration become heightened. The phenomenon described here extends beyond mere migration between urban areas, encompassing movements across various locations, each characterized by its unique assortment of infections. When confronted with armed conflicts, the act of residents being forced to leave their houses extends beyond the simple act of moving their personal belongings. Many individuals may unknowingly be unintentionally carrying germs. These imperceptible hitchhikers can materialize in several manifestations, encompassing viruses, bacteria, or parasites. It is important to note that certain infections can cause evident diseases, whereas others can exist within hosts without producing obvious symptoms, thus, enabling their transmission to other areas and populations without detection.

Armed conflict's consequences reach beyond the immediate and obvious destruction, impacting critical industries such as healthcare. Healthcare systems in areas affected by conflicts have seen significant disruptions. The fundamental components of medical support, such as hospitals, clinics, and other healthcare facilities, confront twofold obstacles: some have been destroyed, while others contend with an immense surge in injured individuals. Consequently, the typical rhythm of healthcare delivery experiences a substantial modification. Health checks, commonly regarded as the initial means of protection against diseases, tend to be overshadowed [3].

Similarly, vaccination campaigns, which are crucial in mitigating the occurrence of infectious disease epidemics, may encounter disruptions or may not be executed with the same level of meticulousness as during periods of tranquility [4]. Furthermore, certain preventive healthcare initiatives, which play a crucial role in protecting the well-being of the general population, encounter obstacles. As a result, these systemic disruptions render the populace more susceptible and increase their likelihood of succumbing to various illnesses [5].

The merging of urban and natural ecosystems has become a significant concern in contemporary conflicts. Wars and ongoing conflicts often motivate individuals to pursue safety, leading them to areas that have not been previously impacted by human activity or regions that have mostly functioned as sanctuaries for various wildlife species [6]. As human populations establish themselves in certain geographical areas, they unintentionally infringe upon the ecological niches of various animal species. The recent spatial closeness between human populations and wildlife has resulted in heightened levels of interaction, thus, leading to an augmented susceptibility to zoonotic diseases. Zoonotic spillover events pertain to occurrences in which infections, initially harbored by animals, successfully establish infection in humans. These occurrences are of special significance due to their potential to give rise to unknown diseases against which humans may possess limited immunity. The convergence of human and wildlife habitats, driven by the destructive consequences of warfare, raises the likelihood of such spillover incidents, presenting significant health hazards to displaced communities and perhaps giving rise to more extensive public health emergencies.

The persistent violence has significantly influenced multiple areas, including the crucial field of wildlife observation. Countries affected by recent armed conflicts are renowned for their diverse and abundant fauna and have historically served as a fertile environment for the emergence and transmission of zoonotic illnesses. The importance of ongoing and comprehensive surveillance of wildlife health is underscored by zoonotic illnesses, which can be transmitted from animals to people. Moreover, understanding the complex movement patterns exhibited by animals and diving into their habits yields vital information that might proactively anticipate and manage the dangers associated with zoonotic outbreaks [7]. Unfortunately, many ongoing conflicts have presented many obstacles that impede the pursuit of these crucial academic endeavors. The capacity of researchers and professionals to regularly observe and assess animal health and behavior has been significantly hindered by the prevailing instability in these regions [8]. The interruption of surveillance systems has the potential to result in undetected risks, ultimately leading to unanticipated occurrences of zoonotic outbreaks. Hence, the ramifications of the battle transcend immediate human casualties and geopolitical implications, presenting an underlying peril to public health as it potentially facilitates the uncontrolled proliferation of zoonotic diseases.

As animals expand their range into previously unexplored habitats, many of them are increasingly encountering human populations in closer proximity. Given the challenging circumstances, it is imperative to comprehend and recognize the potential hazards linked to zoonotic illnesses inside areas affected by armed conflicts. It is imperative to adopt a diverse approach while implementing prevention techniques. On the one hand, increased surveillance of humans and animals must be increased. Mobile health units can play a significant role in ensuring that people get the necessary vaccinations and health checks. Conversely, it is imperative to undertake initiatives to impart knowledge to the general populace regarding the potential hazards of intimate interactions with fauna. Implementing basic preventive measures such as refraining from ingesting wild animals, adhering to proper hygiene practices, and promptly seeking medical assistance upon the onset of symptoms can significantly contribute to preventing outbreaks.

## 3. The Potential Role of Anthropic Factors in Increasing the Risk of Zoonotic Diseases During Some Recent Conflicts

According to the World Organization for Animal Health (WAOH), 75% of newly discovered illnesses are zoonotic—they come from domestic or wild animals. This underscores how vital it is for authorities in charge of animal health and those concerned with public health to work together closely [9]. Most recent armed conflicts have far-reaching effects on public health, the environment, and human lives, in addition to international politics [10]. For example, the Ukraine–Russia conflict has been ongoing for more than 10 years and is characterized by military clashes, political unrest, and humanitarian crises [10].

The conflicts in Sudan and in Gaza have resulted in thousands of deaths, injuries, and the displacement of people [11]. This mass displacement of populations, often to overcrowded camps with poor sanitation, increases human–animal contact and creates ideal conditions for the transmission of zoonotic diseases. Disruption of food systems and reduced access to healthcare further heighten the risk of disease outbreaks. Conflicts also damage veterinary infrastructure and services, hampering the ability to monitor animal health and control diseases that could spill over into human populations. Comprehensive surveillance of both human and animal health is critical to anticipate and manage the risks of zoonotic disease transmission in conflict-affected regions like Sudan [11, 12].

Szewczyk et al. [13] reported that the Ukraine conflict has caused significant ecological damage, including damage to infrastructure and the Chernobyl exclusion zone, releasing radioactive substances and posing risks to wildlife. The displacement of human populations and lack of maintenance have increased wildlife–human interactions. Ukraine's ongoing war severely impacts the agricultural sector, causing farmers to face challenges and food shortages, leading some communities to seek alternative food sources and potentially increasing the risk of zoonotic diseases [14]. According to Sandvik [15], the ongoing conflict in Ukraine has led to overcrowded living conditions in refugee camps and urban areas, facilitating the spread of zoonotic illnesses through respiratory pathways or close contact between humans and livestock. Zubach et al. [16] highlighted a rare case of leptospirosis that was reported in a Ukrainian civilian during a conflict involving exposure to gray rats in basements. Despite delayed diagnosis and treatment, the case highlights the importance of vigilance in monitoring emerging infectious diseases in conflict-driven living conditions. Deforestation increases zoonotic disease risk due to habitat destruction and transformation into anthropic ecosystems, causing a significant distraction from natural forest ecosystems [17].

The chaos created by wars makes proper surveillance of animal populations difficult, leading to incidents like contaminated animal vaccines in South Sudan in 2017. The conflict has also forced researchers and veterinary professionals to flee Sudan, hindering their capacity to observe and assess animal health and behavior regularly. This exodus of experts has further exacerbated the challenges in managing the risks of zoonotic disease transmission in the region [18].

## 4. Destruction of Ecosystems During the Recent Wars Increase the Risk of Zoonotic Diseases' Emergence and Transmission

The whole world is suffering from the devastating effects of recent wars. The global economy is facing the most crisis period. The recent wars have brought about huge political and humanitarian crises and especially exerted profound ecological repercussions [19]. This may impact the destruction of ecosystems and increase the risk of zoonotic diseases amidst the migratory waves in Africa and Eastern Europe.

The resultant influx of refugees often destroys infrastructure and habitats, which in turn causes wildlife to flee or adapt to new environments, often making them come in close contact with human and domestic animals. Therefore, wildlife migration can significantly impact human–wildlife interactions because of increased encounters between people and animals in new and unfamiliar settings. These interactions can raise the risk of zoonotic disease spillover, which occurs when a disease that typically affects animals is transmitted to humans. In addition, such interactions may also contribute to the transmission of antimicrobial resistance (AMR). Populations of many rare migratory and endemic species are suffering heavy losses. Many species of birds were forced to abandon their natural habitat and nests and change their usual migration routes. War-related activities, such as deforestation and tunnel excavation, contribute to habitat degradation and fragmentation [20]. This degradation can disrupt natural ecosystems, destabilize food chains, and force wildlife into proximity with food animals, potentially facilitating the transmission of new foodborne pathogens. The altered landscape and ecological stressors stemming from conflict can impact the health of wildlife populations. The industrial or populated area may impact the decline in wild species' richness and habitat [21]. Stress and malnutrition in wildlife can weaken their immune systems, making them more susceptible to infections, including zoonotic diseases. This weakened state may increase the likelihood of disease spillover to humans [22]. Belligerents sometimes take advantage of the chaos of war for poaching and trafficking of animal products [21]. This can lead to increased hunting, trapping, and trading of wildlife, potentially introducing zoonotic pathogens into new regions and markets, where human consumption may expose individuals to novel diseases [23].

## 5. The Impact of Inadequate Sanitation on the Emergence of Zoonotic Diseases During the Recent War

The lack of sanitation for migrants of war in conflict countries can have significant consequences, including increased risks of zoonotic diseases [24]. From a public health perspective, many aspects may be considered, including inadequate sanitation facilities, such as access to clean water and proper toilets, which can spread various diseases [25, 26]. Migrants living in crowded and unsanitary conditions are at a higher risk of contracting and spreading infectious diseases, including respiratory infections, diarrheal diseases, and skin infections. These conditions can affect not only the migrants but also the host communities and neighboring regions with their consequent risk, as demonstrated in previous situations. Many foodborne and waterborne diseases are also zoonotic (e.g., brucellosis and leptospirosis) [27]. It is worth noting that many Russian soldiers may have caught anthrax spores, while excavating trenches in the Zaporozhye region. Additionally, there are many unburned anthrax-infected livestock burials in Ukraine. Any digging in these areas will spread illness not just among the occupants but also among the neighboring people.

War and displacement often disrupt ecosystems and may lead to closer contact between humans and animals, increasing the risk of zoonotic disease transmission [28–30]. Poor sanitation practices and lack of clean water can exacerbate this risk, leading to contaminated water sources, improper disposal of animal waste, and increased exposure to disease vectors like insects and arthropods [31–33]. Inadequate sanitation and hygiene can also lead to issues with food safety. Improper handling, storage, and food preparation can result in foodborne illnesses, which can be particularly dangerous when limited or overwhelmed by healthcare and medical facilities [34, 35].

The lack of access to basic sanitation facilities can also have a detrimental effect on the mental and emotional well-being of migrants. It can lead to stress, anxiety, and insecurity as people live in unsanitary and overcrowded conditions. Living in such conditions increases zoonotic disease risk [36, 37]. Access to clean water and sanitation is considered a basic human right. The lack of these essential services can strip migrants of dignity, exacerbate their hardships due to conflict and displacement, and lead to waterborne diseases, including zoonoses. It can also contribute to feelings of marginalization and social exclusion [38, 39].

Poor sanitation and the associated health risks can have long-term implications for the health of migrants. This includes the potential for chronic health problems, developmental issues in children, and increased mortality rates. Addressing the lack of sanitation and mitigating the risks of zoonotic diseases among migrants in conflict countries is crucial for their well-being, public health, and regional stability. Humanitarian organizations and governments should work together to provide clean water, adequate sanitation facilities, and education on hygiene practices to mitigate these risks and improve the overall living conditions of migrants. Additionally, efforts to address zoonotic disease risks may involve increased surveillance, vaccination programs for animals, and public health campaigns to minimize contact between humans and potentially infected animals [40–43].

## 6. Impacts of Recent Wars on the Emergence of Diseases

Several diseases have reemerged as a result of the recent conflicts, such as cholera, tuberculosis, hepatitis, poliomyelitis, measles, human immunodeficiency virus (HIV), and multidrug-resistant (MDR) bacteria. In this section, we will shed light on the impact of recent conflicts on these diseases.

### 6.1. Cholera Outbreaks

Cholera is one of the most common diseases associated with conflicts [44, 45], hence, the transmission is caused most likely by a severe shortage of clean water, unsafe food, inadequate sanitation, overcrowding, and basic healthcare services. A higher incidence of cholera outbreaks has been reported during recent conflicts in Sudan and Syria [46, 47]. In December 2022, a total of 5105 cholera cases were reported across Lebanon, attributed to the population displacement after the Syrian crisis [48] via pollution from untreated sewage and the environment (rivers and irrigation channels) [49].

### 6.2. Tuberculosis

The incidence of tuberculosis increased during the Ukraine–Russia conflicts as a result of displacement and migration [50, 51]. Additionally, drug-resistant *Mycobacterium tuberculosis* has been reported in both Ukraine and Russia [51], as well as other countries such as France [52]. Indeed, the interruption of tuberculosis treatment during conflicts is a major risk for increased drug resistance. Gebreyohannes et al. [52] reported on the impacts of armed conflicts on tuberculosis burden and treatment outcomes. Although six studies reported an increase in tuberculosis during conflicts, three studies reported overall decreases in tuberculosis case notifications. The authors summarized the main reasons for the emergence of tuberculosis during conflicts as follows: (i) destruction of the healthcare system; (ii) malnutrition, mass migration, and poor living conditions, and (iii) overcrowding that are worsened during conflicts.

### 6.3. Poliomyelitis

Several studies reported an increase in poliomyelitis cases in countries with conflict and instability [53, 54]. The increase in poliomyelitis could be attributed to the reduced rates of polio vaccination and disruption in vaccine coverage [55]. In 1999, Gaza was declared free of polio, however, in 2024, circulating vaccine-derived poliovirus type 2 was confirmed in environmental (wastewater) samples that were collected from two different sites in Gaza (Khan Younis and Deir al Balah). More recently, in August 2024, poliomyelitis was reported in a 10-month-old infant suffering from partial paralysis [56]. It is recommended to implement both vaccination and hygienic measures since the virus can be transmitted through the fecal–oral route.

### 6.4. Measles

Measles is one of the most contagious diseases, causing clinical disease in nonimmune people. Overcrowding, inadequate hygienic measures, low vaccination coverage, limited healthcare infrastructure, difficulties in accessing healthcare services, lack of awareness of the benefits of vaccination, and malnutrition are the most important risk factors for infection [57, 58]. An increased incidence of measles was reported in conflict-affected countries, including Sudan [59] and Nigeria [60].

### 6.5. HIV

Several studies reported that HIV is associated with conflict [61–64]. Several risk factors could impact the emergence of HIV, including multiple traumas during wars, financial constraints, interruption of the health system, and lack of diagnostics. Three surveys have been conducted in 31 destination countries to have information on the number of refugees from Ukraine receiving ART in their country. A total of 6519 refugees (1.5 per 1000 refugees) received ART, lower than previous estimates by WHO, ECDC, and partners of between 0.16% and 1.0%. It was proposed that these discrepancies suggest a substantial number of undiagnosed and/or diagnosed, but untreated HIV infections, posing a high risk of disease transmission. In Germany, 46 refugees with HIV from Ukraine were examined, most of them had undetectable HIV viral load. 50.4% of these patients exhibited antibodies against hepatitis B virus (HBV) infection without evidence of replication. Antibodies against hepatitis C virus (HCV) were detected in 23 refugees, but only 10 patients had been diagnosed with HCV previously. Detectable HCV-RNA was evident in nine patients, while 16 refugees had a positive tuberculosis interferon-gamma [65, 66]. Health challenges posed by war migration extend beyond HIV to coinfections such as HBV, HCV, and tuberculosis [66].

### 6.6. AMR

Wars disrupt socioeconomic factors such as trade, travel, demographics, poverty, and cultural practices, influencing the dynamics of zoonotic diseases. Overcrowding of individuals and poor sanitary conditions in conflict areas can lead to the emergence of several pandemics, such as cholera, measles, meningitis, malaria, pneumonia, and tuberculosis [67]. Additionally, wars disrupt preventive measures and health care, such as vaccination programs and intervention measures. Additionally, in Ukraine, the surveillance program of food has been impaired [68]. War wounds (weapon wounds) can also be a significant source of MDR bacteria. Studies on military personnel in the Middle East consistently report the prevalence and patterns of antibiotic resistance in infections resulting from combat-related injuries [69–72]. Over the past two decades, cases of antimicrobial-resistant microorganisms in people with war wounds have been reported in military conflicts in Iraq and Afghanistan [73]. Infection of war wounds [74] can be a cause of the emergence of drug resistance due to the use of antibiotics without control and prior sensitivity testing, particularly from the combat zone and temporary evacuation echelon facilities [75]. There is also a potential for colonization with MDR microorganisms due to movement through different levels of the evacuation chain [76]. Indeed, the use of broad-spectrum antibiotics during transportation to the hospital may save lives, but lead development of resistance during subsequent treatment [77]. Additionally, unsanitary conditions, overcrowding in confined areas, and higher population movement increase the risk of pathogens transmission [78, 79].

Currently, there is a lack of data about the actual impacts of the ongoing wars on the emergence of antibiotic resistance. However, several studies reported the emergence of antibiotic resistance associated with the Ukrainian war. MDR *Acinetobacter baumannii* and *Klebsiella pneumoniae* should be considered among other MDR bacteria in injured patients in Ukraine [80]. Additionally, *A. baumannii*, *K. pneumoniae*, *Enterococcus faecium*, and three distinct *Pseudomonas aeruginosa* strains were isolated and characterized from an injured soldier in Ukraine. These isolates were resistant to most antibiotics and carried an array of carbapenemases (*bla*IMP-1, *bla*NDM-1, *bla*OXA-23, *bla*OXA-48, and *bla*OXA-72) and 16S methyltransferases (*arm*A and *rmt*B4) [77]. The negative impacts of the Ukrainian war are not restricted to Ukraine. In Germany, there has been an increase in *K. pneumoniae* producing NDM-1 and NDM-1/OXA-48 since March 2022, coinciding with the arrival of refugees and evacuated patients from Ukraine [81]. In 2022, 34 MDR-Gram-negative isolates were isolated from 17 of 103 Ukrainian patients at the University Hospital Frankfurt, Germany [82]. In the Netherlands, MDR organisms have emerged in patients from Ukraine. Genomic analysis revealed that 60% of isolates contain New Delhi metallo-*β*-lactamase genes [83].

## 7. Conclusions

The migratory wave in Eastern Europe amidst the war in conflict countries presents a multifaceted challenge from a humanitarian and political perspective regarding its ecological implications. The destruction of ecosystems, wildlife displacement, habitat degradation, and altered disease dynamics contribute to an elevated risk of zoonotic disease emergence and transmission in the region. Urbanization, deforestation, climate change, and intensive agricultural practices continue to shape the landscape of zoonotic disease emergence and spread (Figure 1). As the global population grows and food demand increases, the intensification of agriculture and the expansion of livestock production systems create new occasions for pathogen transmission. These systems can also expose workers and nearby residents to heightened health risks. Awareness, surveillance, and sustainable practices are imperative to mitigate these risks. In this ever-evolving world, where human activities increasingly impact the natural environment, understanding the factors driving zoonotic diseases is crucial for preventing and managing outbreaks. A collaborative effort involving public health authorities, environmental agencies, and the agricultural sector is essential to protect human and animal health and maintain the delicate balance of our interconnected ecosystems in general, particularly in countries affected by wars.

## Data Availability

The data supporting this review are from previously reported studies and datasets, which have been cited. The processed data are available from the corresponding author upon request.

## References

[1] Sikkema R. S., Koopmans M. P. G. (2021). Preparing for Emerging Zoonotic Viruses. *Encyclopedia of Virology*.

[2] Rahman M. T., Sobur M. A., Islam M. S. (2020). Zoonotic Diseases: Etiology, Impact, and Control. *Microorganisms*.

[3] Valand P., Miles J., Pandya A. N. (2020). The Deleterious Effects of War and Conflict on the Provision of Health Care for Vulnerable Populations and the Potential Effects of COVID-19 on Vulnerable Populations in Conflict Zones. *International Journal of Surgery: Global Health*.

[4] Raslan R., El Sayegh S., Chams S., Chams N., Leone A., Hajj Hussein I. (2017). Re-Emerging Vaccine-Preventable Diseases in War-Affected Peoples of the Eastern Mediterranean Region—An Update. *Frontiers in Public Health*.

[5] Hauser D. J., Schwarz N. (2020). The War on Prevention II: Battle Metaphors Undermine Cancer Treatment and Prevention and Do Not Increase Vigilance. *Health Communication*.

[6] Massingham E., Almila E., Piret M. (2023). War in Cities: Why the Protection of the Natural Environment Matters even when Fighting in Urban Areas, and What can be Done to Ensure Protection. *International Review of the Red Cross*.

[7] Gaynor K. M., Fiorella K. J., Gregory G. H. (2016). War and Wildlife: Linking Armed Conflict to Conservation. *Frontiers in Ecology and the Environment*.

[8] Tedla M. G., Berhe K. F., Grmay K. M. (2023). The Impact of Armed Conflict on Animal Well-Being and Welfare, and Analyzing Damage Assessment on the Veterinary Sector: The Case of Ethiopia’s Tigray Region. *Heliyon*.

[9] Usatine R. P., Yosef T., Laplante M., Ankad B. S., Fernandes E. L., Parasitoses E. E. (2023). *Clinical and Dermoscopic Atlas of Non-Neoplastic Dermatoses: Variability According to Phototypes*.

[10] Hryhorczuk D., Levy B. S., Prodanchuk M. (2024). The Environmental Health Impacts of Russia’s War on Ukraine. *Journal of Occupational Medicine and Toxicology*.

[11] Afriyie F. A. (2024). Untangling Sudan’s Discord: Decrypting the Intricate Threads of Turmoil. *The Journal of Intelligence, Conflict, and Warfare*.

[12] Musa M. K., Eshun G., Modber M. A. A. (2024). Public Health Consequences of Armed Conflict in Sudan in the Face of Global Donor Fatigue. *Public Health Challenges*.

[13] Szewczyk T., Werszko J., Slivinska K., Laskowski Z., Karbowiak G. (2021). Molecular Detection of *Bartonella* spp. in Rodents in Chernobyl Exclusion Zone, Ukraine. *Acta Parasitologica*.

[14] Ben Hassen T., El Bilali H. (2022). Impacts of the Russia-Ukraine War on Global Food Security: Towards More Sustainable and Resilient Food Systems?. *Foods*.

[15] Sandvik K. B. (2023). The Ukrainian Refugee Crisis: Unpacking the Politics of Pet Exceptionalism. *International Migration*.

[16] Zubach O., Pestushko I., Dliaboha Y., Semenyshyn O., Zinchuk A. (2023). A Single Clinical Case of Leptospirosis in a 70-Year-Old Man During the Military Conflict in Ukraine. *Vector-Borne and Zoonotic Diseases*.

[17] Sehgal R. N. M. (2010). Deforestation and Avian Infectious Diseases. *Journal of Experimental Biology*.

[18] Mohammed A. A. A., Ahmed M. (2023). Veterinary Services Under Siege: How the Armed Conflict in Sudan Threatens Animal and Human Health and How to Respond. *Infection Ecology & Epidemiology*.

[19] Rawtani D., Gupta G., Khatri N., Rao P. K., Hussain C. M. (2022). Environmental Damages due to War in Ukraine: A Perspective. *Science of the Total Environment*.

[20] Pereira P., Bašić F., Bogunovic I., Barcelo D. (2022). Russian-Ukrainian War Impacts the Total Environment. *Science of the Total Environment*.

[21] Shuaib Y. A. (2024). Zoonotic Disease Vulnerability Escalates amid Sudan’s Armed Conflict. *The Lancet*.

[22] Boyle S. F., Corrigan V. K., Buechner-Maxwell V., Pierce B. J. (2019). Evaluation of Risk of Zoonotic Pathogen Transmission in a University-Based Animal Assisted Intervention (AAI) Program. *Frontiers in Veterinary Science*.

[23] Wilkie D. S., Godoy R. A. (2001). Income and Price Elasticities of Bushmeat Demand in Lowland Amerindian Societies. *Conservation Biology*.

[24] Goto R., Pinchuk I., Kolodezhny O., Pimenova N., Skokauskas N. (2023). Study Protocol: Adolescents of Ukraine During the Russian Invasion (AUDRI) Cohort. *BMC Public Health*.

[25] Quintero K., Durán C., Duri D. (2012). Household Social Determinants of Ascariasis and Trichuriasis in North Central Venezuela. *International Health*.

[26] Coswosk É. D., Neves-Silva P., Modena C. M., Heller L. (2019). Having a Toilet Is Not Enough: The Limitations in Fulfilling the Human Rights to Water and Sanitation in a Municipal School in Bahia, Brazil. *BMC Public Health*.

[27] Bonilla-Aldana D. K., Trejos-Mendoza A. E., Pérez-Vargas S. (2023). A Systematic Review and Meta-Analysis of Bovine Brucellosis Seroprevalence in Latin America and the Caribbean. *New Microbes and New Infections*.

[28] Braam D. H., Jephcott F. L., Wood J. L. N. (2021). Identifying the Research Gap of Zoonotic Disease in Displacement: A Systematic Review. *Global Health Research and Policy*.

[29] Cantor D., Swartz J., Roberts B. (2021). Understanding the Health Needs of Internally Displaced Persons: A Scoping Review. *Journal of Migration and Health*.

[30] Villamizar-Peña R., Gutiérrez-Ocampo E., Holguin-Rivera Y. (2021). Leishmaniasis Among Internally Displaced People of Colombia, 2007–2018—A Comparative Analysis With the General Population. *Travel Medicine and Infectious Disease*.

[31] Mukherjee A., Duttagupta S., Chattopadhyay S. (2019). Impact of Sanitation and Socio-Economy on Groundwater Fecal Pollution and Human Health Towards Achieving Sustainable Development Goals Across India From Ground-Observations and Satellite-Derived Nightlight. *Scientific Reports*.

[32] Kong Y.-L., Anis-Syakira J., Fun W. H., Balqis-Ali N. Z., Shakirah M. S., Sararaks S. (2020). SocioEconomic Factors Related to Drinking Water Source and Sanitation in Malaysia. *International Journal of Environmental Research and Public Health*.

[33] Haywood L. K., Kapwata T., Oelofse S., Breetzke G., Wright C. Y. (2021). Waste Disposal Practices in Low-Income Settlements of South Africa. *International Journal of Environmental Research and Public Health*.

[34] Calderón-Villarreal A., Schweitzer R., Kayser G. (2022). Social and Geographic Inequalities in Water, Sanitation and Hygiene Access in 21 Refugee Camps and Settlements in Bangladesh, Kenya, Uganda, South Sudan, and Zimbabwe. *International Journal for Equity in Health*.

[35] Garsow A. V., Campbell E., Closs G., Kowalcyk B. B. (2021). Food Safety Challenges in Refugee Camps: What Do We Know?. *Journal of Food Protection*.

[36] El Arab R. A., Somerville J., Abuadas F. H., Rubinat-Arnaldo E., Sagbakken M. (2023). Health and Well-Being of Refugees, Asylum Seekers, Undocumented Migrants, and Internally Displaced Persons Under COVID-19: A Scoping Review. *Frontiers in Public Health*.

[37] Andersson L. M. C., Hjern A., Ascher H. (2018). Undocumented Adult Migrants in Sweden: Mental Health and Associated Factors. *BMC Public Health*.

[38] Kitamura A., Jimba M., McCahey J. (2018). Health and Dignity of Palestine Refugees at Stake: A Need for International Response to Sustain Crucial Life Services at UNRWA. *The Lancet*.

[39] Mngadi L. C., Cuadros D. F., Tanser F., Burns J. K., Slotow R., Tomita A. (2023). Water, Sanitation and Depression in Rural Communities: Evidence From Nationally Representative Study Data in South Africa. *Psychology, Health & Medicine*.

[40] Ng Q. X., De Deyn M. L. Z. Q., Loke W., Yeo W. S. (2020). Yemen’s Cholera Epidemic Is a One Health Issue. *Journal of Preventive Medicine and Public Health*.

[41] Essar M. Y., Matiashova L., Tsagkaris C., Vladychuk V., Head M. (2022). Infectious Diseases Amidst a Humanitarian Crisis in Ukraine: A Rising Concern. *Annals of Medicine & Surgery*.

[42] Eneh S. C., Admad S., Nazir A. (2023). Cholera Outbreak in Syria Amid Humanitarian Crisis: The Epidemic Threat, Future Health Implications, and Response Strategy–A Review. *Frontiers in Public Health*.

[43] Alawa J., Alawa N., Coutts A., Sullivan R., Khoshnood K., Fouad F. M. (2020). Addressing COVID-19 in Humanitarian Settings: A Call to Action. *Conflict and Health*.

[44] Al-Tammemi A. B., Sallam M. (2023). The Current Cholera Menace Amid the War Crisis in Syria and the Economic Crisis in Lebanon: A Time for Global Solidarity. *New Microbes and New Infections*.

[45] Alhaffar M. H. D. B. A., Gomez M. M. M., Sigua J. A., Eriksson A. (2023). The Cholera Outbreak in Syria: A Call for Urgent Actions. *IJID Regions*.

[46] Jones F. K., Wamala J. F., Rumunu J. (2020). Successive Epidemic Waves of Cholera in South Sudan Between 2014 and 2017: A Descriptive Epidemiological Study. *The Lancet Planetary Health*.

[47] Ahmed S. H., Nashwan A. J. (2022). Cholera Outbreak Amid Civil War: A Public Health Crisis in Syria. *Journal of Infection and Public Health*.

[48] Helou M., Khalil M., Husni R. (2023). The Cholera Outbreak in Lebanon: October 2022. *Disaster Medicine and Public Health Preparedness*.

[49] Kassem I. I., Osman M., Jaafar H., Omari K. E. (2022). Refugee Settlements, Sewage Pollution, COVID-19 and the Unfolding Cholera Outbreak in Lebanon. *Journal of Travel Medicine*.

[50] Dahl V., Migliori G. B., Lange C., Wejse C. (2022). War in Ukraine: An Immense Threat to the Fight Against Tuberculosis. *European Respiratory Journal*.

[51] Guthmann J. P., Fraisse P., Bonnet I., Robert J. (2023). Active Tuberculosis Screening Among the Displaced Population Fleeing Ukraine, France, February to October 2022. *Eurosurveillance*.

[52] Gebreyohannes E. A., Wolde H. F., Akalu T. Y., Clements A. C. A., Alene K. A. (2024). Impacts of Armed Conflicts on Tuberculosis Burden and Treatment Outcomes: A Systematic Review. *BMJ Open, Mar*.

[53] Marou V., Vardavas C. I., Aslanoglou K. (2024). The Impact of Conflict on Infectious Disease: A Systematic Literature Review. *Conflict and Health*.

[54] Akil L., Ahmad H. A. (2016). The Recent Outbreaks and Reemergence of Poliovirus in War and Conflict-Affected Areas. *International Journal of Infectious Diseases*.

[55] Mohamed A., Akbar I. E., Chaudhury S. (2022). Progress Toward Poliomyelitis Eradication ― Afghanistan, January 2021–September 2022. *MMWR. Morbidity and Mortality Weekly Report*.

[56] Zayed D., Banat M., Al-Tammemi A. B. (2024). Infectious Diseases Within a War-Torn Health System: The Re-Emergence of Polio in Gaza. *New Microbes and New Infections*.

[57] Waldman R., Steinglass R., Nieburg P. (2024). An Urgent Case for Measles Vaccination in Gaza. *The Lancet*.

[58] Lanke R., Chimurkar V. (2024). Measles Outbreak in Socioeconomically Diverse Sections: A Review. *Cureus*.

[59] Centers for Disease Control and Prevention (CDC) (2004). Emergency measles control activities—Darfur, Sudan, 2004. *MMWR. Morbidity and Mortality Weekly Report*.

[60] Babakura B., Nomhwange T., Jean Baptiste A. E. (2021). The Challenges of Insecurity on Implementing Vaccination Campaign and its Effect on Measles Elimination and Control Efforts: A Case Study of 2017/18 Measles Campaign in Borno State, Nigeria. *Vaccine*.

[61] Daw M. A., El-Bouzedi A. H., Ahmed M. O. (2022). The Impact of Armed Conflict on the Prevalence and Transmission Dynamics of HIV Infection in Libya. *Frontiers in Public Health*.

[62] Vasylyeva T. I., Liulchuk M., Friedman S. R. (2018). Molecular Epidemiology Reveals the Role of War in the Spread of HIV in Ukraine. *Proceedings of the National Academy of Sciences*.

[63] Katamba A., Ogwang M. D., Zamar D. S. (2020). Cango Lyec (Healing the Elephant): HIV Incidence in Post-Conflict Northern Uganda. *EClinicalMedicine*.

[64] Ali A., Nisar M., Idrees M. (2012). Prevalence of HBV Infection in Suspected Population of Conflict-Affected Area of War Against Terrorism in North Waziristan FATA Pakistan. *Infection, Genetics and Evolution*.

[65] Ahrenstorf G., Dopfer-Jablonka A., Joean O. (2024). Status of HIV and Comorbidities in Refugees With HIV from Ukraine. *HIV Medicine*.

[66] van Bremen K., Parczewski M., Monin M. (2024). HIV Care in Ukrainian Migrants in Two European Countries: All the Same?. *Pathogens*.

[67] Roberts B. (2022). *Issues in Public Health: Challenges for the 21st Century*.

[68] Boiko O., Garkavenko T., Musiiets I., Nedosekov V., Kozytska T. (2024). Salmonellosis in Ukraine: An Analysis of Food Products Contamination, Salmonella Transmission, and Serovar Diversity During 2012–2023. *German Journal of Veterinary Research*.

[69] Murray C. K. (2008). Infectious Disease Complications of Combat-Related Injuries. *Critical Care Medicine*.

[70] Mende K., Stewart L., Shaikh F. (2019). Microbiology of Combat-Related Extremity Wounds: Trauma Infectious Disease Outcomes Study. *Diagnostic Microbiology and Infectious Disease*.

[71] Tribble D. R., Murray C. K., Lloyd B. A. (2019). After the Battlefield: Infectious Complications Among Wounded Warriors in the Trauma Infectious Disease Outcomes Study. *Military Medicine*.

[72] Weintrob A. C., Murray C. K., Xu J. (2018). Early Infections Complicating the Care of Combat Casualties From Iraq and Afghanistan. *Surgical Infections*.

[73] Murray C. K., Yun H. C., Griffith M. E. (2009). Recovery of Multidrug-Resistant Bacteria From Combat Personnel Evacuated From Iraq and Afghanistan at a Single Military Treatment Facility. *Military Medicine*.

[74] Melwani M. (2022). How War is Spreading Drug Resistant Superbugs Across Ukraine and Beyond. *BMJ*.

[75] Loban’ G., Faustova M., Dobrovolska O., Tkachenko P. (2023). War in Ukraine: Incursion of Antimicrobial Resistance. *Irish Journal of Medical Science*.

[76] Valentine K. P., Viacheslav K. M. (2017). Bacterial Flora of Combat Wounds From Eastern Ukraine and Time-Specified Changes of Bacterial Recovery During Treatment in Ukrainian Military Hospital. *BMC Research Notes*.

[77] Mc Gann P. T., Lebreton F., Jones B. T. (2023). Six Extensively Drug-Resistant Bacteria in an Injured Soldier, Ukraine. *Emerging Infectious Diseases*.

[78] Garry S., Checchi F. (2020). Armed Conflict and Public Health: Into the 21st Century. *Journal of Public Health*.

[79] Murray C. J. L., King G., Lopez A. D., Tomijima N., Krug E. G. (2002). Armed Conflict as a Public Health Problem. *BMJ*.

[80] European Food Safety Authority (2022). The European Union One Health 2021 Zoonoses Report. *EFSA journal. European Food Safety Authority*.

[81] Sandfort M., Hans J. B., Fischer M. A. (2022). Increase in NDM-1 and NDM-1/OXA-48-Producing *Klebsiella pneumoniae* in Germany Associated With the War in Ukraine, 2022. *Eurosurveillance*.

[82] Schultze T., Hogardt M., Velázquez E. S. (2023). Molecular Surveillance of Multidrug-Resistant Gram-Negative Bacteria in Ukrainian Patients, Germany, March to June 2022. *Eurosurveillance*.

[83] Zwittink R. D., Wielders C. C., Notermans D. W. (2022). Multidrug-Resistant Organisms in Patients From Ukraine in the Netherlands, March to August 2022. *Eurosurveillance*.

