# Corrigendum to “Migratory Wave due to Conflicts: Risk of Increased Infection From Zoonotic Diseases”

**DOI:** 10.1155/tbed/9868493

**Published:** 2025-06-28

S. S. Tazerji, P. M. Duarte, R. Gharieb, et al., “Migratory Wave due to Conflicts: Risk of Increased Infection From Zoonotic Diseases,” *Transboundary and Emerging Diseases* 2025, no. 1 (2025): 1-9, https://doi.org/10.1155/tbed/5571316.

In the article titled “Migratory Wave due to Conflicts: Risk of Increased Infection From Zoonotic Diseases,” there was a spelling error in author Sina Montajeb's name in the author list, where Sina Montajeb should have read Sina Montajab and affiliated to Department of Clinical Sciences, Faculty of Veterinary Medicine, Tehran University, Tehran, Iran which is incorrect.

The correct affiliation for this author is:

*Faculty of Veterinary Medicine and Science*, *Shahid Bahonar University*, *Kerman*, *Iran*

We apologize for this error.

